# Expression and Pathogenic Analysis of Integrin Family Genes in Systemic Sclerosis

**DOI:** 10.3389/fmed.2021.674523

**Published:** 2021-07-20

**Authors:** Dan Xu, Ting Li, Ruikang Wang, Rong Mu

**Affiliations:** ^1^Department of Rheumatology and Immunology, Peking University Third Hospital, Beijing, China; ^2^Department of Rheumatology and Immunology, Peking University People's Hospital, Beijing, China

**Keywords:** integrin, systemic sclerosis, skin, bioinformatics analysis, gene

## Abstract

**Objectives:** Emerging evidence shows that integrin members are involved in inflammation and fibrosis in systemic sclerosis (SSc). This study aimed at evaluating the expression of integrin family genes in the skin tissue from SSc patients and exploring the potential pathogenic mechanism.

**Methods:** We utilized the public datasets of SSc skin tissue from the Gene Expression Omnibus (GEO) database to analyze the expression and clinical significance of integrin family genes in SSc. The expression of integrin members in skin tissue was also assessed by immunohistochemistry. In addition, functional enrichment and pathway analysis were conducted.

**Results:** Compared with healthy controls, the mRNA and protein levels of *ITGA5, ITGB2*, and *ITGB5* were upregulated in the skin of SSc patients. Further analysis indicated that the mRNA expression levels of *ITGA5, ITGB2*, and *ITGB5* were positively correlated with modified Rodnan skin thickness score (mRSS). Functional enrichment and pathway analysis showed that integrin members may play multiple roles in the pathogenesis of SSc. Among them, *ITGA5, ITGB2*, and *ITGB5* might synergistically promote SSc through affecting extracellular matrix (ECM) turnover, ECM–receptor interaction, focal adhesion, and leukocyte trans-endothelial migration, while *ITGA5* and *ITGB5* also might affect angiogenesis and endothelial cell function. In addition, *ITGA5, ITGB2*, and *ITGA5* were associated with different pathways, respectively. *ITGA5* was uniquely enriched for actin organization, while *ITGB5* was for TGF-β signaling and *ITGB2* for immune cell activation.

**Conclusion:** Our results implied that the abnormal expression of integrin family genes including *ITGA5, ITGB2*, and *ITGB5* may participate in multiple pathological processes in SSc. Further investigations are required for confirming this speculation.

## Introduction

Systemic sclerosis (SSc) is an immune-mediated autoimmune disease, which is characterized by activation of the immune system, dysfunction of endothelial cells, and organ fibrosis (1, 2). The aberrant accumulation of extracellular matrix (ECM) components often disrupts the physiological architecture and leads to angiogenesis and organ fibrosis (3). It has been implied that the interaction between the surrounding ECM, fibroblasts, and endothelial cells is mediated by the expression of specific integrins on the cell surface, which strongly affects cell function (4).

Integrins are a large family of heterodimeric transmembrane receptors, comprising α and β subunits. There are 18 α subunit genes (*ITGA1, ITGA2, ITGA3, ITGA4, ITGA5, ITGA6, ITGA7, ITGA8, ITGA9, ITGA10, ITGA11, ITGA2B, ITGAD, ITGAE, ITGAL, ITGAM, ITGAV, ITGAX*) and 8 β subunit genes (*ITGB1, ITGB2, ITGB3, ITGB4, ITGB5, ITGB6, ITGB7, ITGB8*), forming 24 different transmembrane surface receptors in vertebrates (5). Integrins are widely distributed on cell surfaces of normal fibroblast, endothelial cells, epithelial cells, and immune cells. Integrin changes conformation and activates subsequent intracellular signals by recognizing specific extracellular ligands, so as to mediate cell–cell adhesion and cell–matrix adhesion, respond to microenvironment signals, and regulate cell migration and proliferation (6). Emerging evidence shows that integrins also play important roles in autoimmunity (7), angiogenesis (8), and tissue fibrosis (9) by controlling local activation of latent transforming growth factor (TGF)-β, cross-talking with growth factor receptors (10), mediating mechanotransduction (11), and promoting cell adhesion and proliferation (12).

*ITGA5, ITGB1, ITGB3*, and *ITGB5* are well-studied integrin members in SSc, which can form α5β1, αvβ3, and αvβ5 heterodimeric receptors. Among them, integrin αVβ3 and integrin αVβ5 were found overexpressed in SSc patients and animal models, which were related to lymphocyte infiltration, TGF-β activation, and collagen accumulation (13). On the other hand, studies on other integrin members still remain sparse. Gene analysis revealed that inflammation mediated by the integrin signaling pathway was a common shared pathway related to SSc (14). All these discoveries made integrin an attractive therapeutic target for SSc. Neutralizing antibody and small molecule inhibitors of integrin αV and β1 were protective in animal models of SSc (13, 15, 16). However, integrin-targeted therapy has not shown significant efficacy in patients yet. Phase II clinical trials of an αVβ6 antibody (BG00011) in the treatment of idiopathic pulmonary fibrosis (IPF) and an integrin αV monoclonal antibody (abituzumab) in the treatment of SSc-ILD were terminated due to safety and efficacy problems or difficulties in patient inclusion, respectively (clinicaltrials.gov identifier NCT03573505 and NCT02745145). The failure of the above clinical trials may be partly due to the complexity of the integrins in the progression of SSc. This study aimed to explore the key members and roles of integrins in the pathogenesis of SSc, therefore providing insight into its potential value as a therapeutic target for SSc.

## Methods

### Microarray Data Acquisition and Processing

To explore the changes of expression profiling and reveal biological processes in the skin of patients with SSc, eligible microarray datasets were downloaded from the Gene Expression Omnibus (GEO, http://www.ncbi.nlm.nih.gov/geo) database. The inclusion criteria of microarray datasets were as follows: (1) datasets should be the expression profile of genome-wide mRNA transcriptome data, (2) datasets consisted of expression profiles in the skin of patients with SSc and normal controls, and (3) datasets were standardized or raw datasets. All gene expression data have previously been published on the GEO database.

### Identification of Differentially Expressed Integrin Family Genes

Normalization and log2 transformation were performed for the raw data to minimize heterogeneity. Probes without gene symbols or genes with more than one probe were removed or averaged, respectively. The merged data were preprocessed by sav package to remove batch effects with R software (version 3.5.1). After performing batch normalization, limma package was utilized to screen differentially expressed integrin family member genes between SSc patients and healthy controls. Adjusted *p* < 0.05 was considered statistically significant. The correlations between mRNA expression of integrin members and clinical characteristics were analyzed.

### Immunohistochemistry (IHC)

Immunohistochemical analysis was performed on 3-mm sections of formalin-fixed, paraffin-embedded tissue. Antigen retrieval was performed by a microwave oven with buffer of citric acid (pH 6.0). Then, endogenous peroxidase activity and non-specific binding were blocked with peroxidase block buffer and 1% bovine serum albumin, respectively. After blocking, sections were incubated with primary antibodies (anti-integrin α5 antibody, 1:400, ab150361, Abcam, Cambridge, MA, USA; anti-integrin α7 antibody, 1:800, ab203254, Abcam, Cambridge, MA, USA; anti-integrin β2 antibody, 1:2,000, ab131044, Abcam, Cambridge, MA, USA; anti-integrin β5 antibody, 1:400, 3629S, CST, Danvers, MA, USA) at 4°C overnight. Subsequently, sections were incubated with HRP conjugate before chromogenic detection using DAB. For the semiquantitative analysis, the H-score method assigned a score of 0–300 to each patient, based on the percentage of cells stained at different intensities (17).

### GO and KEGG Enrichment Analyses

Database for Annotation, Visualization, and Integrated Discovery (DAVID) was applied to perform the Gene Ontology (GO) analysis and Kyoto Encyclopedia of Genes and Genomes (KEGG) pathway enrichment analysis. The integrin family genes showed correlation coefficient (*r*) > 0.5 were analyzed for GO and KEGG enrichment by DAVID. GO analysis included biological processes (BP), cellular components (CC), and molecular functions (MF). The top 10 significant enrichments of BP, CC, and MF (*p* < 0.05 and rank by *p*-value) and KEGG enrichments (*p* < 0.05) were visualized.

### Estimating the Population Abundance of Tissue-Infiltrating Immune and Stromal Cell Populations

The absolute abundance of eight immune and two stromal cell populations were analyzed by Microenvironment Cell Populations-counter (MCP-counter) method (18). In addition, we analyzed the association of integrin members and the population abundance of tissue-infiltrating immune and stromal cell by Spearman's correlation.

## Results

### Gene Expression Datasets of SSc

Three expression datasets were obtained with accession numbers of GSE58095, GSE65536, and GSE95065 from the GEO database (19, 20). Totally, skin gene expression profiles of 89 SSc patients and 61 normal controls were enrolled in this study. The detailed information of the four datasets is displayed in Table 1.

**Table 1:** Studies included in the analysis^a^.

**Dataset**	**GEO accession no**.	**Excluded, no**.	**Included, no**.	**No. of samples**
Assassi et al. (19)	GSE58095	5	97	61 SSc, 36 HC
Guo et al. (20)	GSE65336	68	10	10 SSc
Mantero et al.^#x0002A;^	GSE95065	0	33	18 SSc, 15 HC

*Eighty-nine SSc patients and 61 HC were included.*

*SSc, systemic sclerosis; HC, healthy control.*

a *Arrays were excluded if the samples were not skin biopsies or if the samples were collected at a second time point.*

* *Array profiling data can be obtained from the Gene Expression Omnibus (GEO-NCBI) (GSE95065), which is shared by Mantero JCP. et al. The website is https://www.ncbi.nlm.nih.gov/geo/query/acc.cgi?acc=GSE95065*.

### The Aberrant mRNA Expression of Integrin Genes in SSc Patients

**Figure 1:**
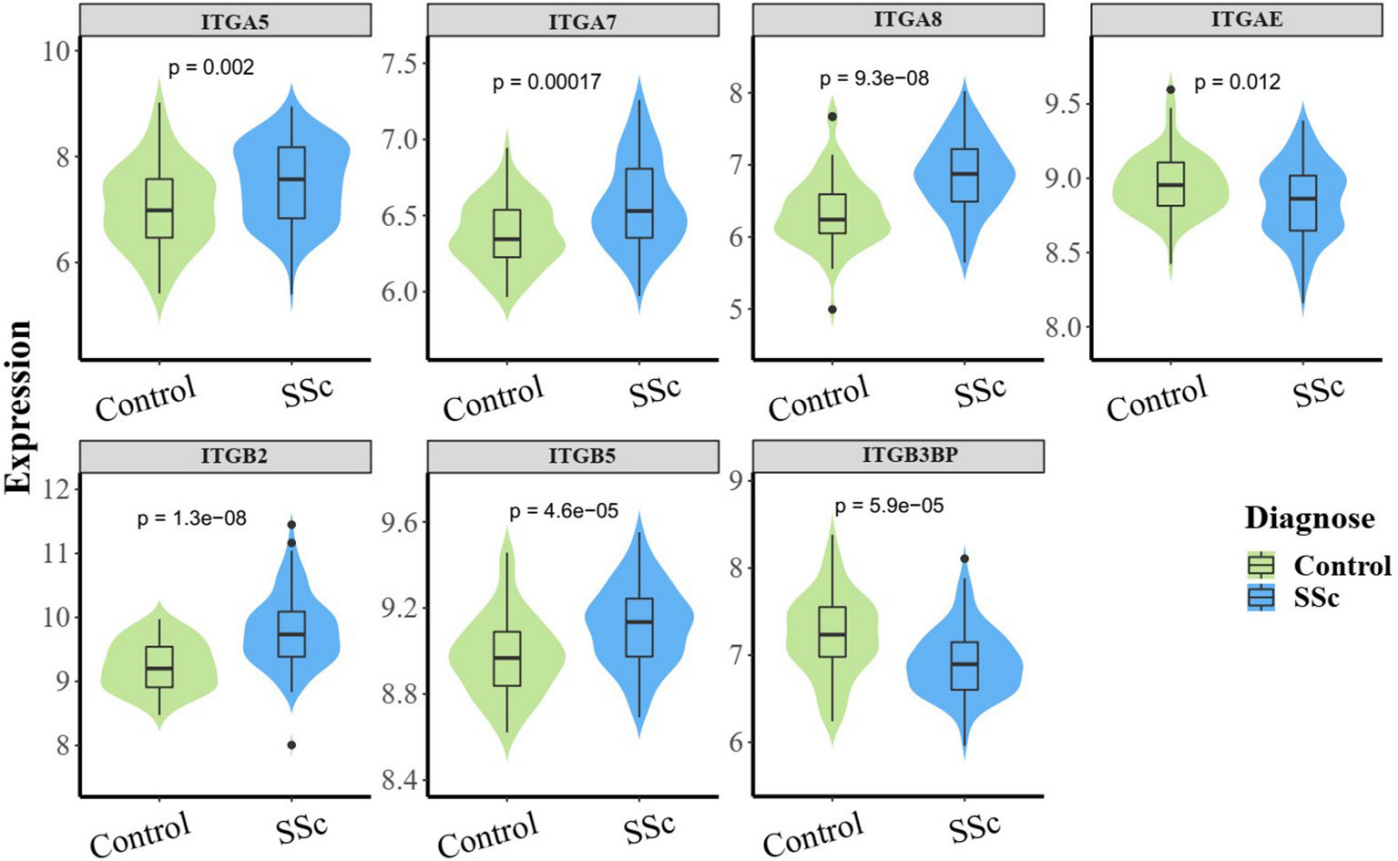
The aberrant mRNA expression of integrin genes in SSc patients. Differentially expressed violin plot of integrin family genes in skin samples of patients with SSc (89 SSc vs. 61 normal control). SSc, systemic sclerosis.

To understand the roles of integrin genes in SSc, we compared the mRNA levels of integrin genes between SSc and normal controls. The results indicated that mRNA levels of *ITGA5, ITGA7, ITGA8, ITGB2*, and *ITGB5* were significantly higher in skin samples of SSc than those of normal controls, while the mRNA levels of *ITGAE* and *ITGB3BP* were significantly lower and other integrins showed no remarkable differences (Figure 1).

### The Protein Levels of Abnormal Integrins in the Skin Tissues of SSc Patients

**Figure 2:**
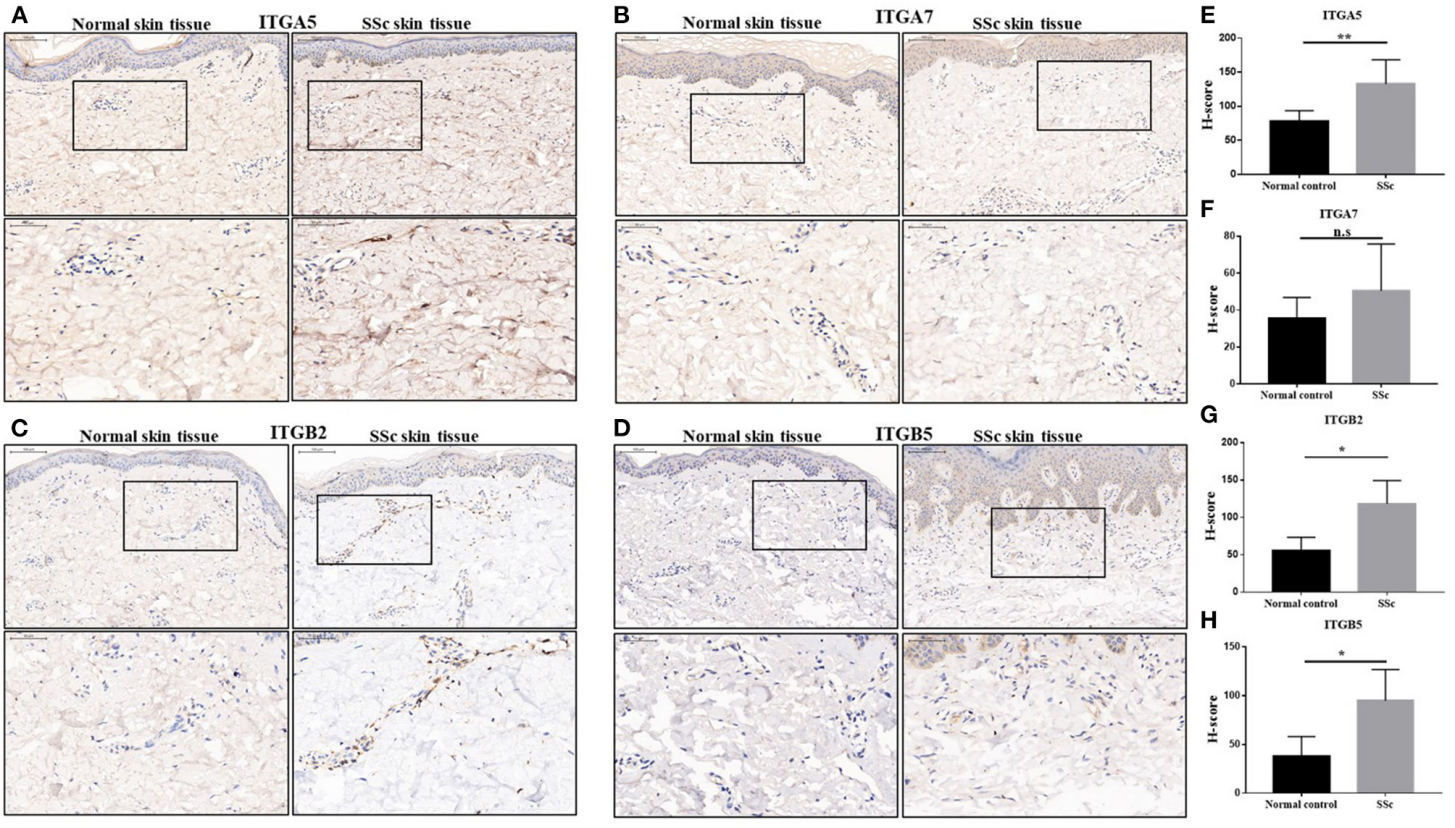
The protein expressions of integrin α5, integrin α7, integrin β2, and integrin β5 in the skin tissues of SSc patients. **(A–D)** Representative images of integrin α5, integrin α7, integrin β2, and integrin β5 immunohistochemical staining in the skin tissues of SSc patients and normal controls. **(E–H)** Comparison of the H-score values of integrin α5, integrin α7, integrin β2, and integrin β5 in SSc patients and normal control. The data are represented as mean ± SD. Statistical differences were performed using the Mann–Whitney test. *p < 0.05, **p < 0.01. SSc, systemic sclerosis.

We next performed an immunohistochemical analysis to evaluate the expression of integrin α5, integrin α7, integrin β2, and integrin β5 in the skin tissue from 10 SSc patients and 6 normal controls. Consistent with the results of gene expression profiles from microarray, the protein levels of integrin α5, integrin β2, and integrin β5 in the skin tissues of SSc patients were significantly increased compared with those in the normal control, respectively. However, the level of integrin α7 was found to have no significant differences between the skin tissues of normal and SSc patients (Figure 2).

### Association Between Aberrant Expressed Integrin Family Genes and Clinical Features of SSc

**Figure 3:**
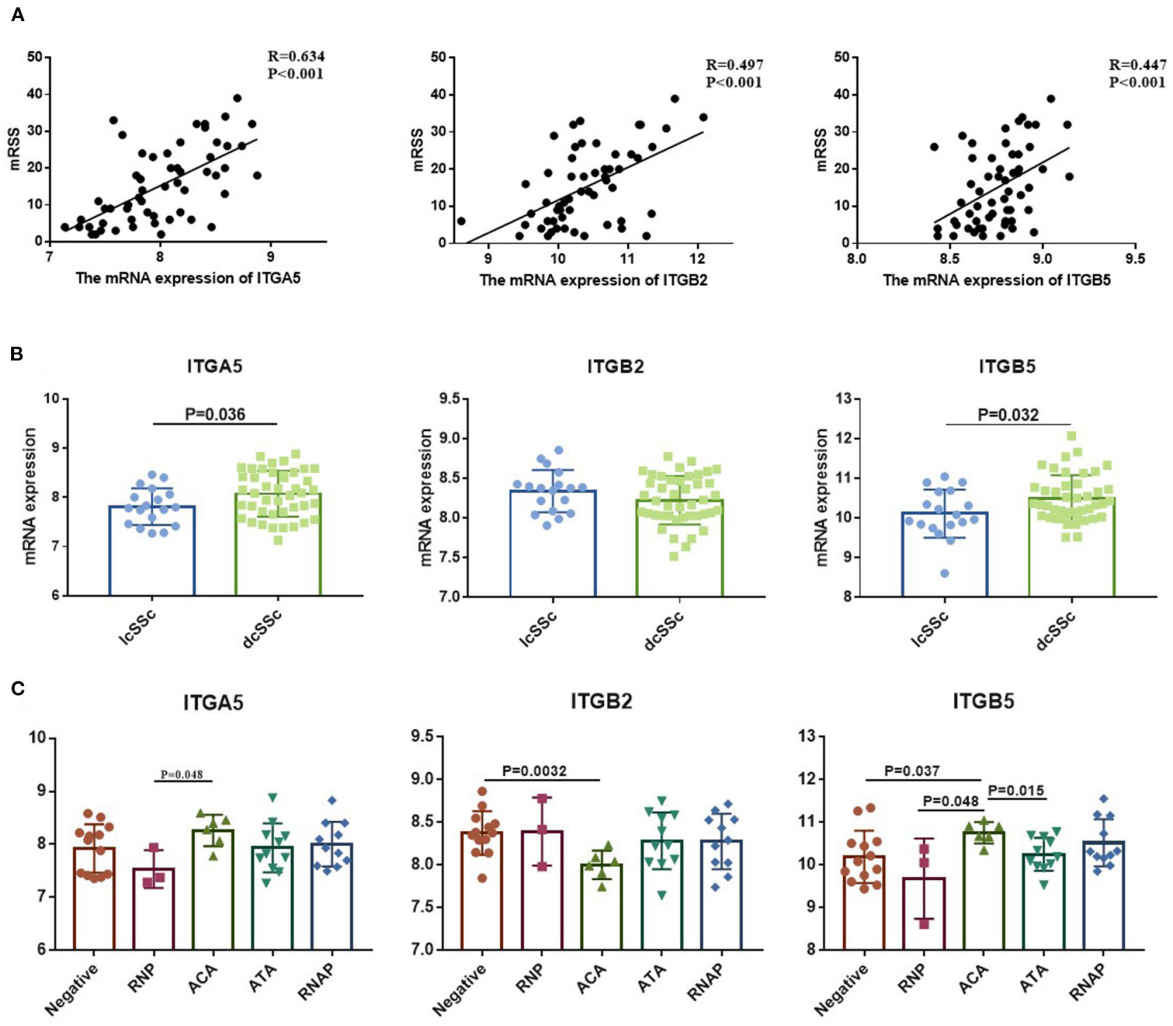
Correlation between aberrant expressed integrin family genes and clinical features of SSc from GSE58095. **(A)** The mRNA expression of *ITGA5, ITGB2*, and *ITGB5* genes was positively correlated with mRSS, respectively. **(B)** The mRNA expression levels of *ITGA5* and *ITGB5* were higher in dcSSc than in lcSSc. **(C)** *ITGB2* was downregulated in patients seropositive for ACA than in lacking specific antibodies. The gene expression of *ITGB5* was higher in patients seropositive for ACA than in those lacking specific antibodies or those seropositive for either RNP or ATA. SSc, systemic sclerosis; dcSSc, diffused cutaneous systemic sclerosis; lcSSc, limited cutaneous systemic sclerosis; mRSS, modified Rodnan skin thickness score; RNP, anti-U1 RNP antibody; ACA, anti-centromere antibody; ATA, anti-topoisomerase antibody; RNAP, anti-RNA polymerase III antibody.

To verify the potential roles of aberrant expressed integrin family genes in SSc, correlation analysis and subgroup analysis between the mRNA levels of *ITGA5, ITGB2*, and *ITGB5* and clinical features were performed in GSE58095. The clinical characteristics of SSc subjects from GSE58095 are concluded in Table 2. We found that the increased *ITGA5, ITGB2*, and *ITGB5* expressions were significantly associated with modified Rodnan skin thickness score (mRSS) (Figure 3A). Concerning the SSc subgroups, the mRNA expression levels of *ITGA5* and *ITGB5* were higher in dcSSc skin tissue than in lcSSc (Figure 3B). We compared integrin family gene expression in the skin sample according to the type of autoantibody in SSc patients, while we observed that *ITGB2* was downregulated in patients seropositive for anti-centromere antibody (ACA) compared with those lacking specific antibodies. We also found that the gene expression of *ITGB5* was higher in patients seropositive for ACA than in those lacking specific antibodies or those seropositive for either anti-U1 RNP antibody (RNP) or anti-topoisomerase antibody (ATA) (Figure 3C).

**Table 2:** Demographic and clinical characteristics of subjects at skin biopsy.

**Characteristics**	**SSc patients (*n* = 61)**	**Controls (*n* = 36)**	***p*-value**
Age median (range), years	54 (22–82)	50 (23–67)	0.025
Gender, female/male, *n* (%)	45/16 (73.77)	29/7 (80.56)	0.448
**Type of SSc**, ***n*** **(%)**			
dcSSc	43 (70.49)		
lcSSc	18 (29.51)		
Total mRSS, median (range)	14 (2–39)		
Interstitial lung disease, *n* (%)	23 (37.70)		
FVC of predicted %, median (range)	76 (35–121)		
DLCO of predicted %, median (range)	61 (23–120)		
**Antibody**			
Negative, *n* (%)	17 (27.87)		
RNP, *n* (%)	3 (4.92)		
ACA, *n* (%)	7 (11.48)		
ATA, *n* (%)	17 (27.87)		
RNAP, *n* (%)	17 (27.87)		
Immunosuppressive agents, *n* (%)	17 (27.87)		

*SSc, systemic sclerosis; dcSSc, diffused cutaneous systemic sclerosis; lcSSc, limited cutaneous systemic sclerosis; mRSS, modified Rodnan skin thickness score; FVC, forced vital capacity; DLCO, carbon monoxide breath diffusion capacity; RNP, anti-U1 RNP antibody; ACA, anti-centromere antibody; ATA, anti-topoisomerase antibody; RNAP, anti-RNA polymerase III antibody*.

### Correlation Between Aberrant Expressed Integrin Family Genes and the Infiltration of Immune and Stromal Cells in the Skin of SSc

**Figure 4:**
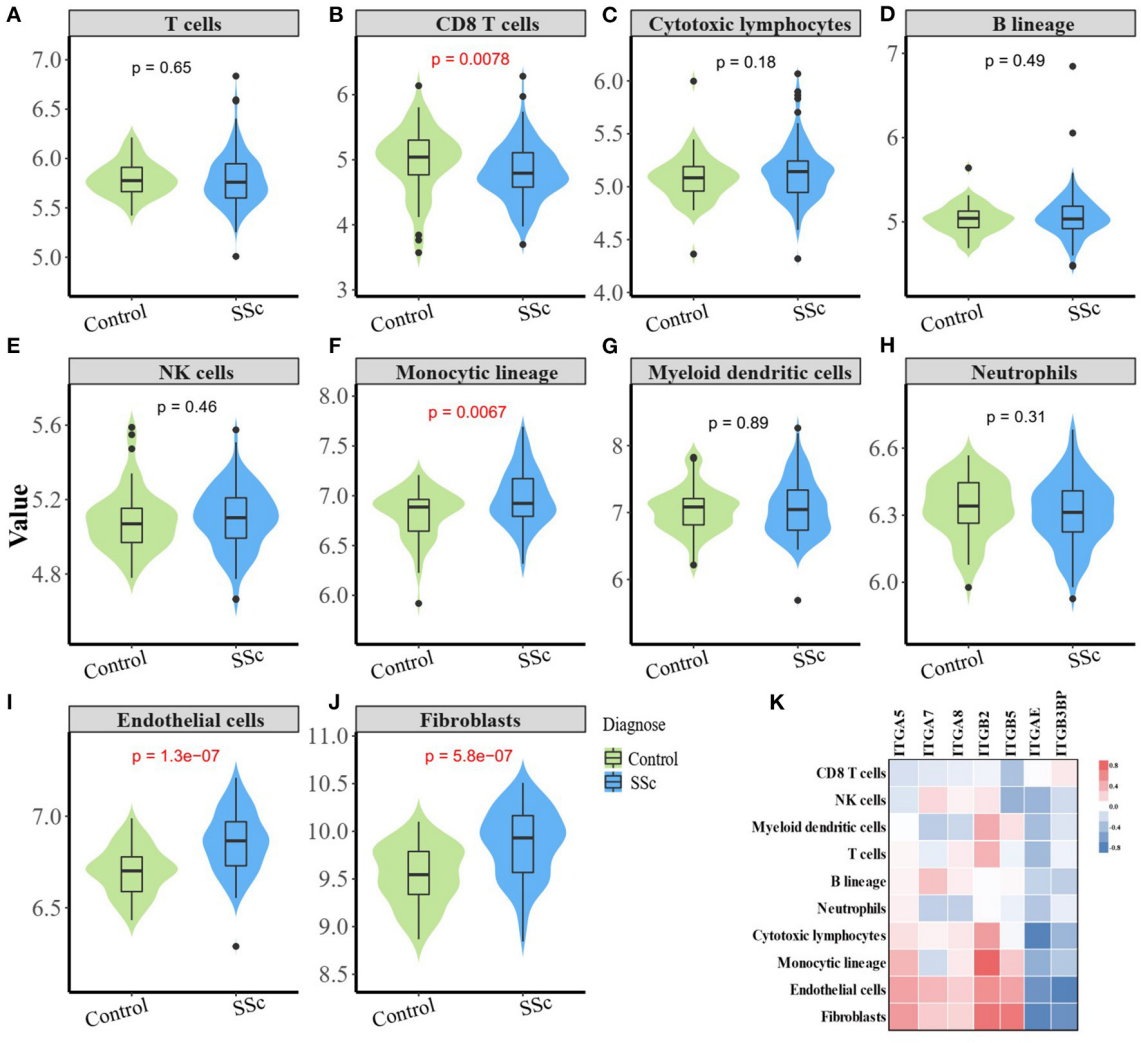
Correlation between aberrant expressed integrin family gene and the infiltration of immune and stromal cells in the skin of SSc. **(B)** The abundance of CD8^+^ T cell is relatively low in the skin from SSc patients than healthy controls. **(F, I, J)** The abundance of monocytic lineage, endothelial, and fibroblast were higher in SSc patients compared with healthy control, respectively. **(A, C–E, G, H)** The abundance of total T cells, cytotoxic lymphocytes, B cells, NK cells, myeloid dendritic cells, and neutrophils showed no difference between SSc patients and healthy control. **(K)** The mRNA expression level of *ITGA5* was positively correlated with fibroblasts (*r* = 0.47, *p* < 0.0001), endothelial cells (*r* = 0.44, *p* < 0.0001), and monocytes (*r* = 0.35, *p* < 0.0001). The mRNA expression level of *ITGB2* was significantly higher than that of fibroblasts (*r* = 0.64, *p* < 0.0001), endothelial cells (*r* = 0.52, *p* < 0.0001), T cells (*r* = 0.36, *p* < 0.0001), and myeloid dendritic cells (*r* = 0.39, *p* < 0.0001). The mRNA expression level of *ITGB5* was positively correlated with fibroblasts (*r* = 0.63, *p* < 0.0001), endothelial cells (*r* = 0.43, *p* < 0.0001), and monocytes (*r* = 0.26, *p* = 0.0021). SSc, systemic sclerosis.

The composition of immune cells, fibroblast, and endothelial cell in the skin of SSc patients and healthy control was analyzed, and the fraction of eight kinds of immune cells was shown in the violin plot (Figures 4A–J). As shown, the abundance of monocytic lineage, endothelial cell, and fibroblast was higher in SSc patients than in healthy controls, while the abundance of CD8^+^ T cell was relatively lower in SSc patients.

The mRNA expression of *ITGA5, ITGB2*, and *ITGB5* showed significant correlation with the abundance of tissue-infiltrating immune cells or stromal cells in the SSc skin sample. Among them, the mRNA expression level of *ITGA5* was positively correlated with the abundance of fibroblasts (*r* = 0.47, *p* < 0.0001), endothelial cells (*r* = 0.44, *p* < 0.0001), and monocytes (*r* = 0.35, *p* < 0.0001), suggesting that *ITGA5* may participate in the pathogenesis of scleroderma through these cells. The mRNA expression level of *ITGB2* was significantly correlated with the abundance of fibroblasts (*r* = 0.64, *p* < 0.0001), endothelial cells (*r* = 0.52, *p* < 0.0001), T cells (*r* = 0.36, *p* < 0.0001), and myeloid dendritic cells (*r* = 0.39, *p* < 0.0001). The mRNA expression level of *ITGB5* was positively correlated with the abundance of fibroblasts (*r* = 0.63, *p* < 0.0001), endothelial cells (*r* = 0.43, *p* < 0.0001), and monocytes (*r* = 0.26, *p* = 0.0021). The detailed correlation between the abundance of immune and stromal cell types and mRNA expression levels of integrin member genes is shown in Figure 4K.

### Possible Mechanisms of *ITGA5, ITGB2*, and *TIGB5* Involved in SSc

**Figure 5:**
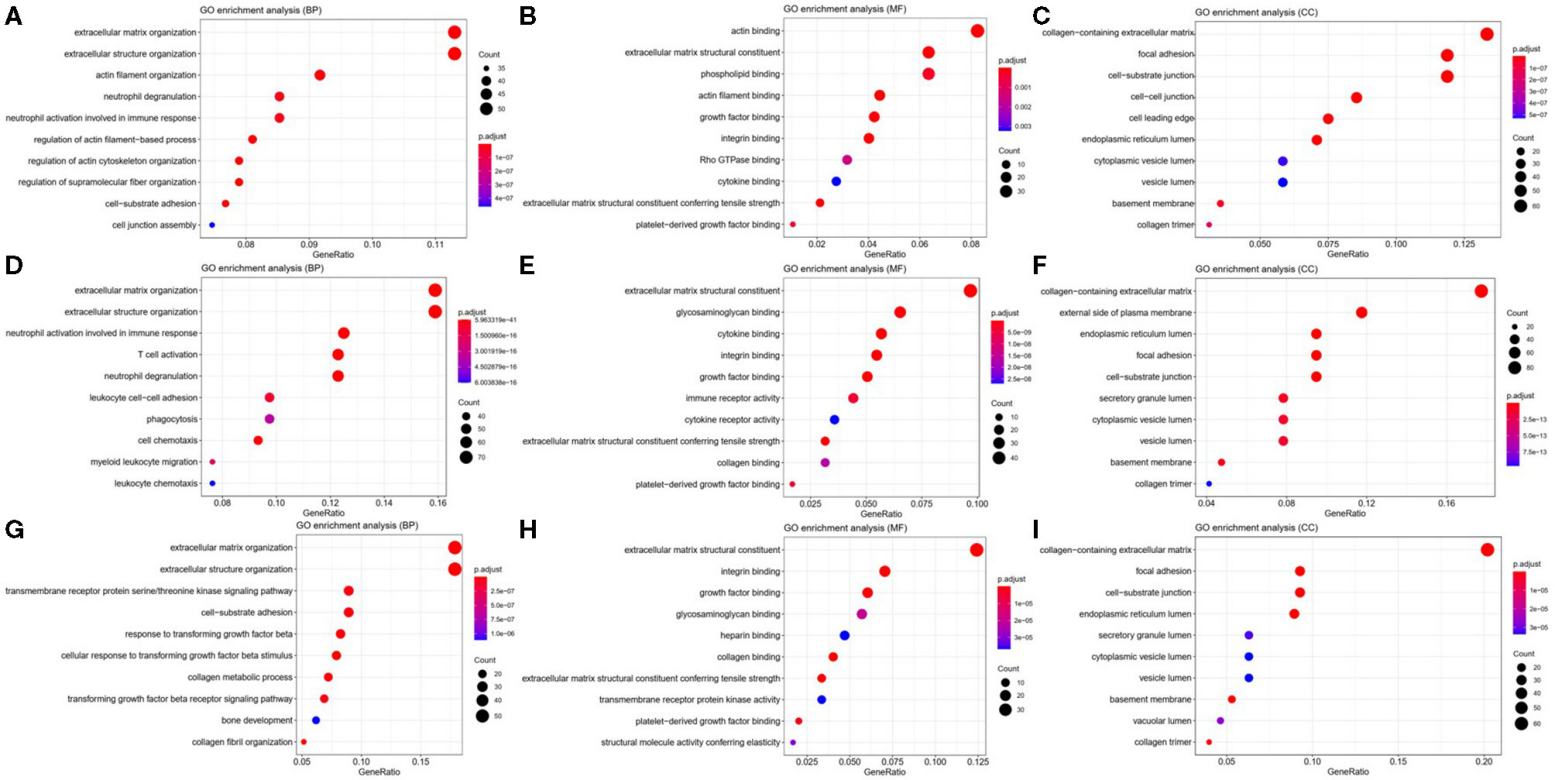
Possible mechanisms of *ITGA5, ITGB2*, and *ITGB5* involved in SSc. Top 10 significantly enriched GO terms for *ITGA5* **(A–C)**, *ITGB2* **(D–F)**, and *ITGB5* **(G–I)**. The size of the circle represents gene count. Different colors of circles represent different adjusted *p*-value. Gene ratio means the number of genes in *ITGA5* and *ITGB2*/*B5* co-expressed genes (*r* > 0.5); genes that belong to this pathway divided by the number of genes in the background gene cluster that belong to this pathway. GO, Gene Ontology; SSc, systemic sclerosis.

**Figure 6:**
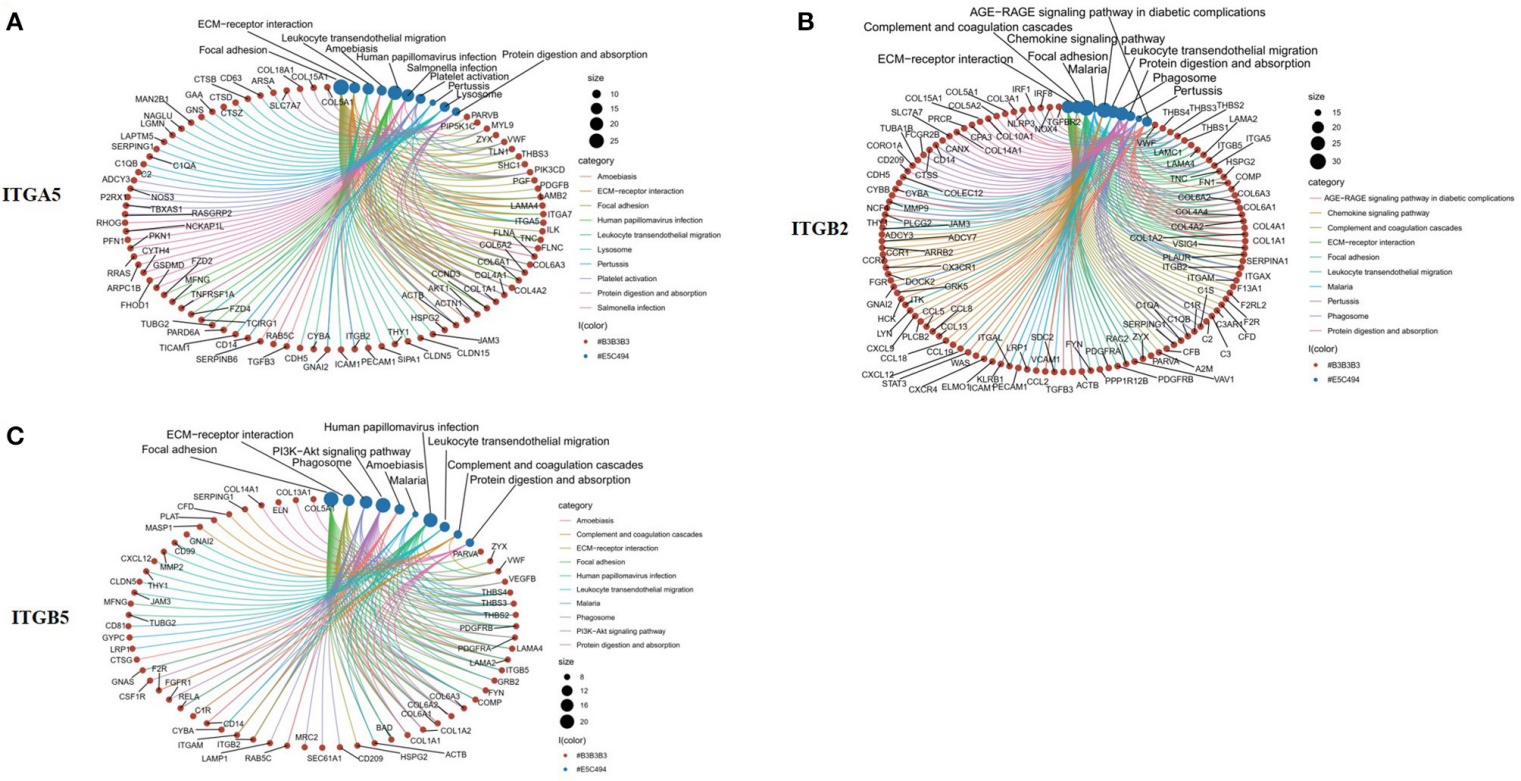
Top 10 significantly enriched KEGG pathway for *ITGA5, ITGB2*, and *ITGB5* involved in SSc. **(A)** ITGA5; **(B)** ITGB2; **(C)** ITGB5. The red circle represents genes, and the blue circle represents related signaling pathways enriched by genes. KEGG, Kyoto Encyclopedia of Genes and Genomes; SSc, systemic sclerosis.

To explore the potential function of *ITGA5, ITGB2*, and *ITGB5* in SSc, their co-expressed genes (*r* > 0.5) were analyzed by GO and KEGG in the DAVID database. There were the same GO terms and KEGG pathway that appeared in *ITGA5, ITGB2*, and *ITGB5* top enrichment lists, including GO:0005201 (extracellular matrix structural constituent), GO:0005178 (integrin binding), GO:0019838 (growth factor binding), GO:0030020 (extracellular matrix structural constituent conferring tensile strength), and GO:0048407 (platelet-derived growth factor binding) in molecular function enrichment; GO:0062023 (collagen-containing extracellular matrix), GO:0031012 (extracellular matrix), GO:0005604 (basement membrane), GO:0005788 (endoplasmic reticulum lumen), GO:0005581 (collagen trimer), GO:0005925 (focal adhesion), and GO:0030055 (cell-substrate junction) in cellular components enrichment; GO:0030198 (ECM organization) and GO:0043062 (extracellular structure organization) in biological processes enrichment (Figures 5A–I); and hsa04510 (focal adhesion), hsa04512 (ECM–receptor interaction), hsa05144 (malaria), hsa04670 (leukocyte trans-endothelial migration), and hsa04974 (protein digestion and absorption) in KEGG enrichment (Figures 6A–C). In summary, *ITGA5, ITGB2*, and *ITGB5* might synergistically promote SSc through the extracellular matrix turnover, ECM–receptor interaction, focal adhesion, and leukocyte trans-endothelial migration. It is worth mentioning that *ITGA5-* and *ITGB5*-associated genes were enriched for GO:0001525 (angiogenesis) in the biological processes enrichment.

In addition, *ITGA5*-associated genes were uniquely enriched for GO:0007015 (actin filament organization), GO:0051493 (regulation of cytoskeleton organization), GO:0032956 (regulation of actin cytoskeleton organization), GO:0031589 (cell-substrate adhesion), GO:1902903 (regulation of supramolecular fiber organization), GO:0030335 (positive regulation of cell migration), and GO:0032970 (regulation of actin filament-based process). The results indicated that *ITGA5* might be more involved in the organization and regulation of actin cytoskeleton, cell adhesion, and migration in SSc. *ITGB2*-associated genes were uniquely enriched for GO:0043299 (leukocyte degranulation), GO:0050900 (leukocyte migration), GO:0006909 (phagocytosis), GO:0002275 (myeloid cell activation involved in immune response), GO:0002444 (myeloid leukocyte mediated immunity), GO:0042110 (T-cell activation), GO:0042119 (neutrophil activation), and GO:0002250 (adaptive immune response). These results implied that *ITGB2* might be important for inflammation by cytokine binding and activation of T cells, monocytes, and granulocytes in SSc. *ITGB5* associated genes uniquely enriched for GO:0032963 (collagen metabolic process), GO:0030199 (collagen fibril organization), GO:0071559 (response to transforming growth factor beta), GO:0071560 (cellular response to transforming growth factor beta stimulus), GO:0007179 (transforming growth factor beta receptor signaling pathway), GO:0007178 (transmembrane receptor protein serine/threonine kinase signaling pathway), and GO:0032964 (collagen biosynthetic process). These results indicated that *ITGB5* might be involved in the transforming growth factor beta signaling pathway.

## Discussion

Integrin plays important and complex roles in SSc, but integrin-targeted therapy has not shown significant efficacy in SSc patients, which may be due to that the precise role of integrin is currently ambiguous and unsuitable option for treatment target. In this study, we used the GEO database to explore the expression and potential roles of integrin family genes in SSc skin, hoping to find a feasible target. We found that the mRNA and protein levels of *ITGA5, ITGB2*, and *ITGB5* were abnormally overexpressed in the skin of SSc. Further analysis indicated that the mRNA levels of *ITGA5, ITGB2*, and *ITGB5* were positively correlated with mRSS score. Besides, we also found that the mRNA levels of *ITGB2* and *ITGB5* were associated with positive autoantibodies. *ITGA5, ITGB2*, and *ITGB5* showed strong correlations with various stromal cells including endothelial cell and fibroblast, respectively.

Mechanically, our results showed *ITGA5, ITGB2*, and *ITGB5* were mainly enriched for ECM and cell–matrix interaction, indicating that they might promote SSc by affecting ECM turnover, ECM–receptor interaction, focal adhesion, and leukocyte trans-endothelial migration. Growth factor, chemokines, ADP, and thrombin can interact with talin and kindlin, called “inside-out” signaling, and promote the activity of integrins (6, 21). On the other hand, integrins, like integrin α2β1 (coded by *ITGA2* and *ITGB1*), can directly bind to GFOGER-like motifs of collagen I to enhance collagen synthesis without the help of inside-out signaling (22). On the other hand, integrins bind to ECM ligands and subsequently trigger the accumulation of complex adaptors and signaling proteins, activating integrin downstream signaling pathways, such as the activation of focal adhesion kinase (FAK), SRC, AKT, and ERK pathways, which is known as the “outside-in” signaling, and consequently regulating cell behavior, such as the inhibition of cell death, the regulation of cytoskeletal dynamics and cellular structure, and intracellular transport or migration (23). In summary, these results indicated that as receptors of ECM components, *ITGA5, ITGB2*, and *ITGB5* participate in the fibrosis process through connecting ECM components with FAK, which led to FAK activation, further promoting the proliferation, activation, and migration of fibroblasts and endothelial cells.

Our study also showed that *ITGA5, ITGB2*, and *ITGB5* may mediate the migration of leukocytes from blood vessels to skin tissue by interacting with chemokines (CXCL12, CCL2) and vascular endothelial cell adhesion molecules (JAM3/CDH5/ICAM 1/PECM1) and participate in the immune inflammatory process of SSc. Overexpression of *ITGB2* can recognize ligands such as intercellular adhesion molecule 1 (ICAM-1) and vascular cell adhesion molecule 1 (VCAM-1) on the surface of endothelial cells, and then provide the direct cellular binding to vascular endothelial cells and fibroblasts, which facilitates the infiltration of inflammatory cells to the endothelium and subsequent transmigration (24). A recent study also showed that interleukin 1β (IL-1β) not only induced inflammatory but also increased the expression of integrin α5β1 to promote trans-endothelial migration of peripheral blood mononuclear cells (PBMC) in human brain microvascular endothelial cells (25). Generally speaking, the interaction between inflammatory cells, endothelial cells, and fibroblasts regulated by integrin family members including *ITGA5, ITGB2*, and *ITGB5* may lead to the activation of vascular endothelial cells and fibroblasts in SSc. Our results indicated that *ITGA5* and *ITGB5* also synergistically affected angiogenesis and endothelial cell function. Wang et al. found that higher matrix stiffness increased VEGFR2 expression in human umbilical vein endothelial cells and that the integrin αVβ5/Akt/Sp1 pathway participated in stiffness-mediated effects on VEGFR2 upregulation (26). Integrin α5β1 levels in endothelial cells were induced in response to several angiogenic factor stimuli, such as IL-8, bFGF, or TNFα. Upregulated integrin α5β1 participated in regulating angiogenesis by interacting with diverse partners such as VEGFR-1, CD97, and uPAR (27–29). In addition, blocking integrin α5 subunits by specific monoclonal antibodies or small peptides has become a potential strategy for anti-angiogenesis therapy (30).

Overall, integrin members have a compensatory and synergistic effect, which may explain why the current therapies targeting integrin have not achieved good efficacy in fibrosis or SSc. Combined therapy targeting multiple integrin members in SSc should be considered.

Our study also suggested that *ITGA5*, IGTB5, and IGTB2 participated in SSc pathogenesis in unique ways. RNA interference high-throughput assay showed that *ITGA5* is one of the genes that affect the myofibroblast phenotype of SSc skin fibroblasts (31). A higher expression level of *ITGA5* was found in the serum-derived fibroblasts of IPF patients than in the normal cells (32), which facilitated a more aggressive proliferative phenotype of fibroblasts (33). This was consistent with our findings that *ITGA5* mRNA expression was positively correlated with fibroblasts, suggesting that *ITGA5* may mainly affect the phenotype of fibroblasts and affect the process of fibrosis. We also found that *ITGA5* was related with cytoskeleton and actin. Supportively, a previous study showed that *ITGA5* can modulate peripheral actin organization (34). Presumably, *ITGA5* participated in cell stiffness and contraction phenotype, while *ITGB5* participated in TGF-β signaling in SSc patients. *ITGB5* combined with αV can bind to arginine-glycine-aspartic acid (RGD) leading to the activation of TGF-β1 and the enhancement of TGF-β signaling by physical association with TGF-βRII (35, 36). Yoshihide et al. found that αVβ5 was upregulated in scleroderma fibroblasts and that the transient overexpression of αVβ5 increased the human alpha2 (I) collagen gene expression in normal fibroblasts (37). *ITGB2* correlated with activation of T cell, neutrophil, and monocyte, indicating that *ITGB2* may be involved in the inflammation of SSc. Physiologically, *ITGB2* was important for host defense, which was exclusively expressed in leukocytes promoting leukocyte adhesion and ensuing extravasation (38). In summary, each integrin has its specific role and function in SSc, which involves many aspects such as immunity, inflammation, and fibrosis, indicating that the integrin family members play a wide and important role. In addition, it also reminds us of the importance of further searching for molecules that can simultaneously regulate the activation of several integrin members such as talin and kindlins (6).

This study has some limitations. The protein levels of integrin α8, integrin αE, and integrin β3 binding protein remain to be detected in the skin tissues of SSc patients. Second, our study only focused on the integrin mRNA expression in skin samples, leaving the expression and function of integrin in PBMC, lung, and other involved tissues unconsidered. Therefore, further studies are necessary to validate our findings and conjectures *in vitro* and *in vivo*.

In conclusion, this study revealed that the mRNA and protein levels of *ITGA5*, IGTB5, and IGTB2 were upregulated in the skin tissue of SSc patients. *ITGA5*, IGTB5, and IGTB2 may participate in multiple pathological processes, and preliminary evidence of existing overlapping mechanisms has been noticed. Further studies are warranted.

## Data Availability Statement

Publicly available datasets were analyzed in this study. This data can be found here: https://www.ncbi.nlm.nih.gov/geo/query/acc.cgi?acc=GSE58095 https://www.ncbi.nlm.nih.gov/geo/query/acc.cgi?acc=GSE65336 https://www.ncbi.nlm.nih.gov/geo/query/acc.cgi?acc=GSE95065.

## Ethics Statement

The studies involving human participants were reviewed and approved by the Ethical Committee of the Peking university people's hospital. The patients/participants provided their written informed consent to participate in this study.

## Author Contributions

RM designed this study. DX, TL, and RW contributed to the data interpretation and analysis. All authors contributed to the drafting and revising of the manuscript and have critically reviewed and approved the final submitted version to be published.

## Conflict of Interest

The authors declare that the research was conducted in the absence of any commercial or financial relationships that could be construed as a potential conflict of interest.
